# Polymerization Kinetics, Shrinkage Stress, and Bond Strength to Dentin of Conventional and Self-adhesive Resin Cements

**DOI:** 10.3290/j.jad.b3441537

**Published:** 2022-09-28

**Authors:** Gabriel Nima, Patricia Makishi, Bruna Marin Fronza, Paulo Vitor Campos Ferreira, Roberto Ruggiero Braga, André Figueiredo Reis, Marcelo Giannini

**Affiliations:** a Associate Researcher, Faculty of Health Sciences, Universidad Científica del Sur, Lima, Peru. Performed the microtensile bond strength experiment, performed the scanning electron microscopy evaluation for the fracture pattern analysis of the interface, performed statistical evaluation and wrote the manuscript.; b Collaborative Researcher, Department of Restorative Dentistry, Piracicaba Dental School, State University of Campinas, Piracicaba, SP, Brazil. Idea, hypothesis, supervision, co-wrote manuscript and contributed substantially to the discussion and review.; c Postdoctoral Researcher, Department of Biomaterials and Oral Biology, School of Dentistry, University of São Paulo, São Paulo, SP, Brazil. Idea, hypothesis, performed the polymerization kinetics and shrinkage stress tests, performed statistical evaluation, and contributed substantially to the discussion and review.; d Dentist, Department of Restorative Dentistry, Piracicaba Dental School, State University of Campinas, Piracicaba, SP, Brazil. Performed the microtensile bond strength experiment, performed the scanning electron microscopy evaluation for the fracture pattern analysis of the interface.; e Associate Professor, Department of Biomaterials and Oral Biology, School of Dentistry, University of São Paulo, São Paulo, SP, Brazil. Helped to perform the shrinkage stress test and contributed substantially to the discussion and review.; f Associate Professor, Department of Restorative Dentistry, College of Dentistry, University of Florida, Gainesville, FL, USA. Helped to perform the polymerization kinetics test and contributed substantially to the discussion and review.; g Associate Professor, Piracicaba Dental School, State University of Campinas, Piracicaba, SP, Brazil. Idea, hypothesis, supervision, contributed substantially to the discussion and review.

**Keywords:** resin cement, degree of conversion, rate of polymerization, gel time, shrinkage stress, microtensile bond strength.

## Abstract

**Purpose:**

To evaluate the kinetics of polymerization and shrinkage stress of resin cements, as well as their bond strength to dentin after 24-h or one-year water storage.

**Materials and Methods:**

Three conventional resin cements were evaluated: RelyX Ultimate (RUL), Panavia V5 (PNV), and Multilink N (MLN); and three self-adhesive resin cements: RelyX Unicem 2 (RUN), Panavia SA Cement Plus (PSA), and G-CEM LinkAce (GCL). Degree of conversion (DC), maximum polymerization rate (RP_max_) and gel time values were obtained using Fourier-transform infrared spectroscopy (FTIR/ATR). Shrinkage stress values were determined with a tensiometer, using a universal testing machine (n=5). Indirect resin composite restorations (Solidex) were fabricated and cemented to the dentin surface using self-adhesive resin cements, or conventional resin cements with self-etching adhesive (n=5). Bonding performance was evaluated with the microtensile bond strength (µTBS) test after 24 h or one year of water storage.

**Results:**

MLN exhibited a higher DC (76.7%), whereas the percentage of other materials differed slightly (ranging from 54% to 58.5%). The RP_max_ and shrinkage stress values differed significantly between the cements. PSA showed the longest gel time. Significantly higher µTBS were observed for conventional resin cements after 24-h and one-year storage; a decrease in µTBS was observed for MLN only.

**Conclusion:**

Self-adhesive resin cements may not perform as well as conventional resin cements. Although both categories of cements presented similar polymerization kinetics and shrinkage values, the self-adhesive resin cements showed lower µTBS compared to those of conventional resin cements. Nevertheless, storage time only affected the bonding performance of MLN.

Resin cements have become reliable materials for indirect restorations owing to their improved mechanical properties and adhesion to different structures in comparison with those of water-based cements.^10^ The cementing procedure is expected to provide an effective bond between the resin cement and tooth structure, which significantly affects the longevity of an indirect restoration.^12,32^ Conventional resin cements are used with etch-and-rinse adhesives or adhesive-containing self-etch primers. Conversely, self-adhesive cements resemble compomers when acid-functionalized monomers are added to demineralize the tooth substrate.^16^

Shrinkage of resin-based materials occurs during polymerization, which is characterized by inevitable volumetric contraction.^5^ The distance between the monomer chains is reduced and the chain is converted into polymers.^15^ During the conversion, the viscosity of the material increases rapidly while fluidity decreases (gel point),^39^ and the resin starts to develop elastic properties.^7,33^ If the internal stress generated within the resin material overcomes its bond strength to the tooth surface, debonding at the restoration-tooth interface may occur.^7,33^ Postoperative sensitivity, marginal debonding, and marginal discoloration are associated with clinical outcomes.^8^ Thus, a high initial bond strength is desirable for the long-term durability of restorations.

The degree of conversion (DC) typically represents the percentage of polymerizable double bonds that are converted into single bonds.^39^ After the polymerization reaction, residual monomers can remain in the cured materials. A low DC may result in a decrease in the resistance to wear and color stability.^39^ The polymerization rate also contributes to the magnitude of internal shrinkage stress,^23^ with higher rates leading to higher stress. Meanwhile, a shorter gel time indicates that the material is more sensitive to light curing, which results in a shorter time to allow the resin to flow and relieve the shrinkage stress.^39^ Regarding the polymerization kinetics, an ideal resin cement would have a low polymerization rate, longer gel time, and consequently, low shrinkage stress.

The material composition, the DC of the resin matrix, and its filler volume fraction are determinants of polymerization shrinkage.^5^ Several dual-cure resin cements have been commercially introduced with varying chemical compositions and bonding strategies to the tooth structure. Owing to these differences, only a few studies have investigated the effect of the delays in light curing^30^ and the effect of light exposure duration,^17^ in an attempt to reduce the polymerization rate and lower the stress. Other studies have focused on the polymerization reaction and the effect of light attenuation caused by the placement of indirect restorations.^24,35^ In addition, the bonding performance of different resin cements to dentin has been widely investigated.^26^ However, there is a lack of information on the polymerization kinetics and shrinkage stress of different categories of resin cements and their influence on the long-term bonding performance to dentin.

In this regard, the aim of this study was to evaluate the degree of conversion (DC), maximum rate of polymerization (RP_max_), gel time, and shrinkage stress of three conventional resin cements and three self-adhesive resin cements. Additionally, the bonding performance of both categories of resin cements to dentin and their interfacial characteristics were assessed after 24 h or 1 year of water storage. The null hypotheses were that (i) there was no significant difference in polymerization kinetics and shrinkage stress among the cements tested, and (ii) there was no difference in bond strength and interfacial characteristics among the cements tested after 24 h or one year of water storage.

## MATERIALS AND METHODS

### Experimental Groups

Six resin cements were evaluated: three conventional resin cements, associated with their respective adhesives (RelyX Ultimate [RUL] with Scotchbond Universal [3M Oral Care; St Paul, MN, USA], Panavia V5 [PNV] with Panavia V5 Tooth Primer [Kuraray Noritake; Tokyo Japan], and Multilink N [MLN] with Multilink N Primer A and Primer B [Ivoclar Vivadent; Schaan, Liechtenstein]); and three self-adhesive resin cements (RelyX Unicem 2 [RUN] [3M Oral Care], Panavia SA Cement Plus [PSA] [Kuraray Noritake], and G-CEM LinkAce [GCL] [GC; Tokyo, Japan]). The chemical composition of the materials used in this study are listed in Table 1. The cements were mixed according to the manufacturers’ instructions using their respective automix tips. For the polymerization kinetics and shrinkage stress evaluations, the conventional resin cements’ pastes were evaluated without their dedicated adhesives. Light curing was performed for 20 s using an LED light-curing unit with wavelength between 390-495 nm and irradiance at 995 ± 2 mW/cm^2^ (VALO, Ultradent; South Jordan, UT, USA).

**Table 1:** Materials used in this study

	Material (batch number), manufacturer	Composition
Conventional resin cement	RelyX Ultimate / A1 (564178) 3M Oral Care; St Paul, MN, USA	TEG-DMA, silane treated glass powder, 2-propenoic acid, 2-methyl-1,1’-[1-(hydroxymethyl)-1,2-ethanediyl]ester, reaction products with 2-hydroxy-1,3-propanediyl dimethacrylate and phosphorus oxide, silane treated silica, oxide glass chemicals, sodium persulfate, tert-butyl peroxy-3,5,5-trimethylhexanoate, acetate monohydrate
	Scotchbond Universal (571695) 3M Oral Care	MDP, bis-GMA, HEMA, 2-propenoic acid, 2-methyl-, reaction products with 1,10-decanediol and phosphorous oxide, ethanol, water, copolymer of acrylic and itaconic acid, camphorquinone, dimethylaminobenzoat(-4)
	Panavia V5 / Clear (1U0001) Kuraray Noritake; Tokyo, Japan	Bis-GMA, TEG-DMA, silanated barium glass filler, silanated fluoroalminosilicate glass filler, colloidal silica, surface treated aluminum oxide filler, hydrophobic aromatic dimethacrylate, hydrophilic aliphatic dimethacrylate, camphorquinone, initiators, accelerators
	Panavia V5 Tooth Primer (6B0003) Kuraray Noritake	MDP, HEMA, hydrophilic aliphatic dimethacrylate, accelerators, water
	Multilink N / Transparent (U14830) Ivoclar Vivadent; Schaan, Liechtenstein	Base: bis-GMA, bis-EMA, HEMA, ytterbium trifluoride, 2-dimethylaminoethyl methacrylate Catalyst: UDMA, bis-EMA, HEMA, ytterbium trifluoride, dibenzoyl peroxide
	Multilink N Primer A + B (U12051/U09377) Ivoclar Vivadent	Primer A: 2,2’-[(4-methylphenyl)imino]bisethanol Primer B: phosphonic acid acrylate, HEMA
Self-adhesive resin cement	RelyX Unicem 2 / A2 (562548) 3M Oral Care	TEG-DMA, glass powder, surface modified with 2-propenoic acid, 2-methyl-, 3-(trimethoxysilyl)propyl ester, phenyltrimethoxy silane, bulk material, 2-propenoic acid, 2-methyl-,1,1’-[1-(hydroxymethyl)-1,2-ethanediyl]ester, reaction produtcs with 2-hydroxy-1,3-propanediyl dimethacrylate and phosphorous oxide, silate treated silica, oxide glass chemicals, sodium persulfate, tert-butyl peroxy-3,5,5-trimethylhexanoate, acetic acid, cooper(2+) salt, monohydrate
	Panavia SA Cement Plus / A2 (1U0001) Kuraray Noritake	Bis-GMA, TEG-DMA, HEMA, sodium fluoride, silanated barium glass filler, silanated colloidal silica, MDP, hydrophobic aromatic dimethacrylate, hydrophobic aliphatic dimethacrylate, dl-camphorquinone, peroxide, accelerator, catalysts
	G-CEM LinkAce / Translucent (1405281) GC; Tokyo, Japan	Paste A: UDMA, dimethacrylate Paste B: UDMA, dimethacrylate, phosphoric acid ester monomer, initiator, stabilizer

Abbreviations: bis-GMA: bisphenol-A diglycidyl dimethacrylate; TEG-DMA: triethyleneglycol dimethacrylate; HEMA: 2-hydroxyethyl methacrylate; UDMA: urethane dimethacrylate; 10-MDP: 10-methacryloyloxydecyl dihydrogen phosphate; bis-EMA: ethyoxylated bisphenol A dimethacrylate.

### Polymerization Kinetics

**Fig 1:**
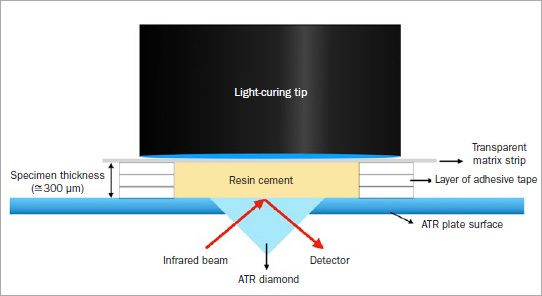
Schematic view of the polymerization kinetics test method used in this study, demonstrating the interaction between the infrared beam and the specimen, cement thickness of approximately 300 µm, a transparent matrix strip on top of the specimen to minimize the presence of an oxygen-inhibited layer, and the light-curing tip.

A Fourier transform near-infrared spectrometer (FTIR; Tensor 27, Bruker; Billerica, MA, USA) with an attenuated total reflectance crystal (ATR; Golden Gate, Specac; Orpington, Kent, UK) was used to monitor the DC of the specimens during the light curing of all groups. The system operated with 4 scans per spectrum, with a resolution of 4 cm^-1^ and a 20-Hz scan rate. Five specimens (n = 5) for each cement were measured. To ensure a standardized cement thickness (approximately 300 µm), 3 layers of adhesive tape (3M Oral Care) were placed around the diamond ATR surface to act as a spacer. Each cement was placed directly onto the diamond window, and the free surface was covered by a transparent matrix strip (Epitex, GC) to minimize the presence of an oxygen-inhibited layer, then gently pressed (Fig 1). Real-time data were obtained from the moment that the light-curing unit was turned on, for a duration of 10 min. The test was performed under 85% relative humidity with controlled room temperature at 23°C.

DC values (%) were obtained by comparing the peak areas in the spectra derived from the uncured and cured resin. Changes in the reaction peak of methacrylate vinyl (aliphatic, at 1635 cm^-1^) were used to follow the polymerization reaction, and those of the symmetric ring stretching (aromatic, at 1610 cm^-1^) were used as internal reference. Due to the fact that UDMA-based resin cements do not contain an aromatic ring, for G-CEM LinkAce, the C-H peak (at 1455 cm^-1^) was used. The DC was calculated according to the equation DC% = (1 – R_cured_/R_uncured_) x 100, where R is the ratio of the aromatic C=C peak (or C-H peak for G-CEM LinkAce) to aliphatic peak areas. The DC represents the proportion of polymerized monomers with the reaction time. The maximum polymerization rate (RP_max_) was calculated as the first derivative of the DC x time curve (%/s).^18^ The gel time was defined as the time period until RP_max_ was reached.^13,39^

### Shrinkage Stress Test

Polymethyl methacrylate (PMMA) rods (6 mm in diameter x 13 mm or 28 mm in length) were used as the bonding substrate for the cements. To optimize light transmission through the rod end, one side of the 13-mm rod was polished with silicon carbide papers and 1-µm diamond paste. The opposite surface of the 13-mm rod and both surfaces of the 28-mm rod were sandblasted with 100-µm aluminum oxide particles (Bio-Art; São Carlos, SP, Brazil). Methyl methacrylate monomer (JET, Artigos Odontologicos Classico; Campo Limpo Paulista, SP, Brazil) was gently applied onto the sandblasted surfaces, coated with unfilled resin (Adper Scotchbond Multi-Purpose Plus, bottle 3, 3M Oral Care), and light cured for 10 s.

**Fig 2:**
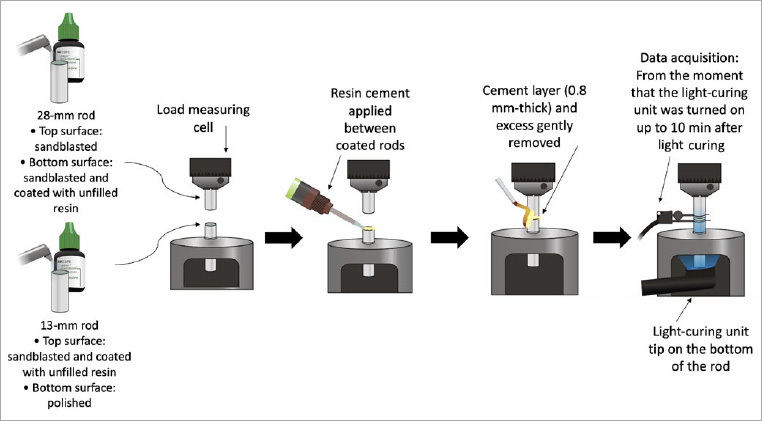
Schematic view of the shrinkage stress test method used in this study. The 13-mm rod and both surfaces of the 28-mm rod were sandblasted with 100-µm aluminum oxide particles. Methyl methacrylate monomer was gently applied onto the sandblasted surfaces, coated with unfilled resin (Adper Scotchbond Multi-Purpose Plus, 3M Oral Care), and light cured for 10 s. The 28-mm rod was attached to an upper clamp, connected to the load cell of a universal testing machine. The 13-mm rod was fixed in a lower clamp to a stainless-steel attachment with a slot allowing the positioning of the light guide in contact with its polished surface. Each resin cement (n = 5) was inserted between the coated surfaces and an actuator was moved downwards to create a cement layer of 0.8 mm thick. The excess cement around the acrylic rod was carefully removed. The specimen thickness was kept constant by the feedback provide from an extensometer. Data acquisition started when the light-curing unit was turned on, until 10 min after light curing.

The 28-mm rod was attached to an upper clamp connected to the load cell of a universal testing machine (Instron 5565, Instron; Norwood, MA, USA). The 13-mm rod was fixed in a lower clamp to a stainless-steel attachment with a slot allowing the positioning of the light guide in contact with its polished surface.^19^ Each resin cement (n = 5) was inserted between the coated surfaces and an actuator was moved downwards to create a cement layer 0.8 mm thick. The excess cement around the acrylic rod was carefully removed. The specimen thickness was kept constant based on the feedback provided from an extensometer (Instron 2630–101, Instron). Data acquisition started 10 s before the light curing was initiated. Values obtained by the load cell corresponded to the force required to compensate the polymerization shrinkage of the cement. Force development was monitored for 10 min (Fig 2), and the maximum load recorded was divided by the respective cross-sectional area of the rod to obtain the maximum stress value in MPa.^19^

### Microtensile Bond Strength (µTBS) Measurement and Fracture Analysis

Resin disks (10 mm diameter x 2 mm thick) were fabricated from indirect resin composite (Solidex, Shofu; Kyoto, Japan) using a silicone mold and light cured for 40 s. One side of the disk was sandblasted perpendicular to the surface using 50-µm aluminum oxide particles (Bio-Art) for 10 s at a 10-mm distance from the tip. The resin disks were ultrasonically cleaned in a water bath for 5 min (SoniClean 2PS, Sanders Medical; Santa Rita do Sapucai, MG, Brazil) and air dried. A coat of RelyX Ceramic Primer (3M Oral Care) was then gently applied, left for 1 min, and an adhesive (Adper Scotchbond Multi-Purpose Plus, bottle 3, 3M Oral Care) was applied.

Thirty freshly extracted, caries-free human third molars were used according to the guidelines of the local Ethics Committee (protocol number 2.057.042). The roots were cut using a low-speed diamond saw (IsoMet, no. 11-4244, Buehler; Lake Bluff, IL, USA) under water coolant. The occlusal enamel was polished with 240-grit silicon carbide paper using a polishing machine (Aropol-E, Arotec; Cotia, SP, Brazil) under running water. The exposed flat dentin surface was wet-polished with 600-grit silicon carbide paper. The dentin substrates were randomly assigned to six groups (n = 5), based on the cementing system used. For conventional resin cements, each cement was applied with its respective self-etching adhesive/primer to dentin, following the manufacturers’ instructions. For the self-adhesive resin cements, no pretreatment of the dentin was performed. The resin cements were applied to the pretreated resin-disk surface prior to the placement on dentin. A constant load of 300 gf was applied during cementation. Excess cement was carefully removed using a microbrush, and 20-s light activation was performed from mesial, buccal, distal, and lingual surfaces to ensure optimum polymerization. After 5 min, the load was removed, and all the surfaces were irradiated for an additional 20 s. An additional 3-mm-thick layer of self-curing composite (Concise, 3M Oral Care) was applied on top of the resin disk to allow easier specimen manipulation during the µTBS test.

After 24-h storage in water in the absence of light at 37°C, the specimens were serially sectioned with a low-speed diamond saw (IsoMet, Buehler) under water cooling to create 0.8 x 0.8 mm^2^ sticks (with the long axis perpendicular to the cavity floor). Half the number of sticks from each tooth were selected randomly for immediate testing, and the remainder were stored for one year, changing the water monthly. For µTBS testing,^2^ the ends of the sticks were fixed with a cyanoacrylate glue (Zapit, DVA; Corona, CA, USA) to a jig in a universal testing machine (EZ Test, Shimadzu; Kyoto, Japan) and then subjected to tensile force at a crosshead speed of 0.5 mm/min. Each tooth was considered a statistical unit; thus, all the values obtained from each tooth were averaged.

After testing, the dentin side of the fractured stick was collected, mounted on brass stubs using carbon adhesive tape, and gold-coated (SCD 050; Bal-tec; Balzers, Liechtenstein). The dentin fracture side of each stick was observed using a scanning electron microscope (SEM; JSM-5600LV, JEOL; Tokyo, Japan) at a magnification of 400X to determine the type of fracture (voltage: 15 kV; stick width: 25–30 nm; working distance: 10–20 mm). The failure mode of each stick was determined and classified by an experienced researcher as follows: failure in adhesive (between resin cement and dentin), cohesive within dentin, cohesive within resin cement, fracture between resin cement-adhesive/primer, and mixed failure.

### Resin Cement-Dentin Interface Evaluation

Two sticks of each material and each storage time were randomly selected and embedded in epoxy resin (EpoxiCure2, Buehler) using a stub mold. The specimens’ surfaces were wet-polished with 400-, 600-, and 1200-grit silicon carbide papers, followed by a polishing cloth with a 1-µm diamond solution (MetaDi Supreme, Buehler). The stubs were demineralized in 20% phosphoric acid for 10 s, deproteinized in 10% NaOCl for 10 min, dehydrated in an ascending ethanol series (30%, 50%, 70%, 90%, and 100% for 20 min per step), and finally immersed in hexamethyldisilazane (Electron Microscope Sciences; Fort Washington, PA, USA) for 10 min. After this chemical dehydration, the stubs were gold-coated and observed using SEM at a magnification of 1600X (voltage: 15 kV; stick width: 25–30 nm; working distance: 10–20 mm).

### Statistical Analysis

The data for RP_max_ (%/s), shrinkage stress (MPa) and µTBS were tested for normality and homoscedasticity using the Shapiro-Wilks and Levene’s test, respectively (α = 0.05). Since the data were normally distributed, the mean RP_max_ (%/s) and shrinkage stress (MPa) values were analyzed using one-way ANOVA with Tukey’s post-hoc test. The µTBS data (MPa) were analyzed using two-way ANOVA with Bonferroni’s post-hoc test. All statistical analyses were performed at a significance level of α = 0.05 using SPSS 21 software (IBM; Chicago, IL, USA) for macOS system.

## RESULTS

### Polymerization Kinetics

**Fig 3:**
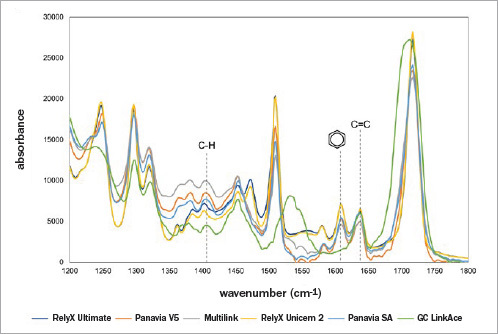
Representative FTIR/ATR spectra of all the resin cements tested.

**Fig 4:**
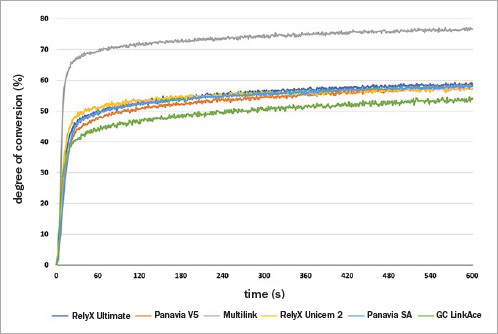
Representative real-time polymerization profile during 10-min analysis of monomer conversion of the resin cements.

The DC, RP_max_, and gel time values are reported in Table 2. Representative FTIR/ATR spectra of all the resin cements tested are shown in Fig 3. For DC, the average nominal value of the resin cements was 56±2%, except for MLN that showed a higher value of 76.7%. Almost immediately after light activation, the DC values increased for all cements. A much slower conversion rate was observed within the remainding 10 min of recording. The real-time conversion profiles of the cements tested are presented in Fig 4. One-way ANOVA showed that the cement used significantly affected the RP_max_ (p = 0.000) and the gel time (p = 0.006).

**Table 2:** Means of degree of conversion (%), maximum polymerization rate (%/s), gel time (s) and shrinkage stress (MPa) of different resin cements

Resin cement	Degree of conversion (%)	RP_max_ (%/s)	Gel time (s)	Shrinkage stress (MPa)
RelyX Ultimate (RUL)	58.5	6.5 (0.9)^bc^	4.2 (1.8)^b^	4.2 (0.2)^a^
Panavia V5 (PNV)	57.9	3.9 (0.6)^d^	5.6 (1.7)^ab^	2.9 (0.6)^b^
Multilink N (MLN)	76.7	12.1 (1.2)^a^	6.6 (1.5)^ab^	4.5 (0.6)^a^
RelyX Unicem 2 (RUN)	57.2	7.2 (1.4)^b^	5.8 (1.7)^ab^	5.0 (0.7)^a^
Panavia SA Cement Plus (PSA)	58.1	4.7 (0.7)^cd^	7.6 (0.5)^a^	4.1 (0.4)^a^
G-CEM LinkAce (GCL)	54.0	8.5 (2.1)^b^	4.4 (0.5)^b^	4.8 (0.6)^a^

Data are presented as the mean (standard deviation). Identical lowercase letters in a column indicate the absence of any statistically significant difference (ANOVA and Tukey’s post-hoc test; p < 0.05).

**Table 3:** Mean microtensile bond strength (MPa) to dentin of different resin cements

Resin cement	Storage time	
	24 h	1 year
RelyX Ultimate (RUL)	37.3 (8.1) ^Aa^	36.9 (4.8) ^Aa^
Panavia V5 (PNV)	42.1 (2.9) ^Aa^	42.0 (2.6) ^Aa^
Multilink N (MLN)	37.7 (3.9) ^Aa^	26.3 (9.7) ^Bb^
RelyX Unicem 2 (RUN)	15.1 (1.5) ^Ab^	12.8 (1.7) ^Ac^
Panavia SA Cement Plus (PSA)	13.4 (2.0) ^Ab^	12.1 (2.9) ^Ac^
GC LinkAce (GCL)	13.2 (1.7) ^Ab^	12.6 (2.8) ^Ac^

Data are presented as the mean (standard deviation). Identical uppercase letters in a row and identical lowercase letters in a column indicate the absence of any statistically significant difference (ANOVA and Bonferroni’s post-hoc test; p < 0.05).

MLN demonstrated significantly higher RP_max_, while lower values were observed for PSA and PNV. No statistically significant difference in RP_max_ was observed between the intermediate values of RUL, RUN, and GCL. Although PSA showed a higher nominal value for gel time, no significant difference was found between PSA, MLN, RUN, and PNV. For RUL and GCL, lower gel times were observed, but neither differed from those of PNV, MLN and RUN.

### Shrinkage Stress

**Fig 5:**
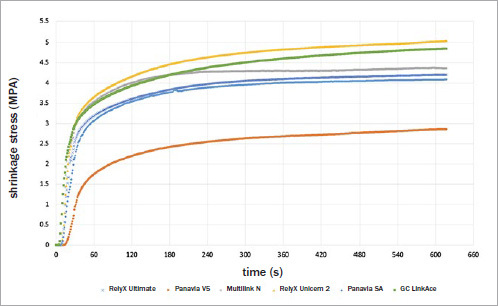
Representative real-time polymerization profile during 10-min analysis of shrinkage stress of the resin cements.

The shrinkage stress values are reported in Table 2, and representative stress development is presented in Fig 5. Although statistically significant differences were found among the materials (p = 0.000), only PNV showed significantly lower shrinkage stress (2.88 ± 0.61 MPa).

### µTBS and Fracture Analysis

Two-way ANOVA of the µTBS revealed statistical differences between cements (p = 0.000) and aging period (p = 0.018); the interaction of these two variables was also significant (p = 0.036). The conventional resin cements (RUN, PNV, and MLN) showed significantly higher bond strengths than those obtained for self-adhesive resin cements (RUL, PSA, and GCL), for both storage times. After 1-year storage, a significant decrease in bonding efficacy was observed only for MLN. The failure mode percentage distribution is summarized in Table 4. The mixed and adhesive failure modes predominated in self-adhesive resin cements for both storage periods. Meanwhile, mixed mode of failure and failure between adhesive/primer-cement predominated for the conventional resin cements at both time points. Among all resin cements tested, only RUL did not present the adhesive mode of failure.

**Table 4:** Distribution (%) of failure modes of the resin cements after different storage periods (24 h / one year)

Resin cement	Adhesive (cement-dentin)	Cohesive within dentin	Cohesive within resin cement	Between cement-adhesive /primer	Mixed
RelyX Ultimate (RUL)	0 / 0	0 / 7	0 / 4	59 / 40	41 / 49
Panavia V5 (PNV)	14 / 4	0 / 2	0 / 0	50 / 54	36 / 40
Multilink N (MLN)	18 / 17	0 / 0	0 / 0	29 / 13	53 / 70
RelyX Unicem 2 (RUN)	59 / 30	0 / 0	0 / 0	0 / 0	41 / 70
Panavia SA Cement Plus (PSA)	75 / 79	0 / 0	0 / 3	0 / 0	25 / 18
G-CEM LinkAce (GCL)	67 / 47	0 / 0	0 / 0	0 / 0	33 / 53


### Resin Cement-Dentin Interface

**Fig 6:**
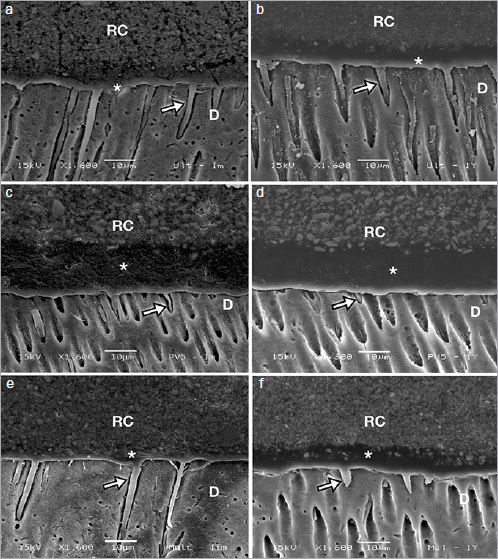
Scanning electron micrographs (1600X) of representative conventional resin cements at the adhesive-dentin interface in RUL, PNV, and MLN after 24-h (a, c, e, respectively) and 1-year storage (b, d, f, respectively). Arrows indicate resin tags. *Adhesive layer. RC: resin cement; D: dentin.

**Fig 7:**
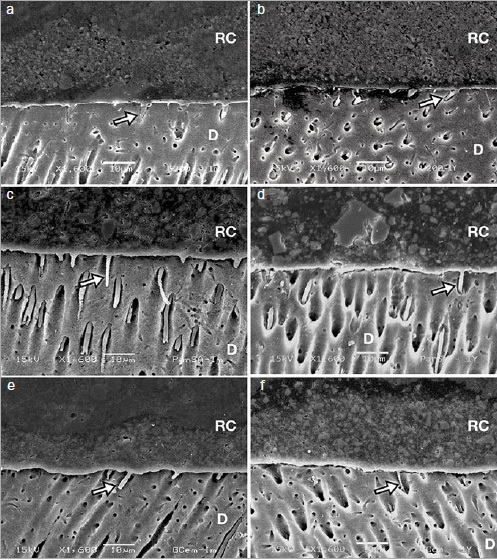
Scanning electron micrographs (1600X) of representative self-adhesive resin cements at the resin cement-dentin interface in RUN, PSA, and GCL after 24-h (a, c, e, respectively) and 1-year storage (b, d, f, respectively). Arrows indicate resin tags. RC: resin cement; D: dentin.

Representative images of resin cement-dentin interface after 24-h or 1-year storage for conventional resin cements and self-adhesive resin cements are shown in Figs 6 and 7, respectively. High-magnification SEM images revealed the presence of resin tags in all materials for both storage times. Conventional resin cements showed thicker resin tags, while thinner resin tags were observed for self-adhesive resin cements.

## DISCUSSION

The present study evaluated the polymerization kinetics and shrinkage stress of conventional resin cements and self-adhesive resin cements, as well as their bonding effectiveness to dentin. The results showed that although a higher DC was observed for MLN, the RP_max_, gel time, and shrinkage stress values varied widely between the cements tested. Thus, the first null hypothesis stating that there was no significant difference in polymerization kinetics and shrinkage stress between the cements tested was rejected, because the polymerization kinetics and shrinkage stress values were affected by the resin cement used. The µTBS results showed that the conventional resin cements obtained significantly higher bond strength than that of the self-adhesive resin cements tested after 24 h or 1 year of water storage. Thus, the second null hypothesis stating that there was no difference in bond strength and interfacial characteristics among the cements tested after 24 h or 1 year of water storage was also rejected.

One concern about resin cements is light attenuation through indirect restorations and insufficient DC.^10,21^ Dual-cure resin cements were developed to optimize the DC in deeper locations by using the combined benefits of light and chemical activation systems.^10^ However, the sole use of chemical activation may result in lower DC compared with that achieved by light activation or when a delay in light activation occurs.^4,28^ The rationale behind delayed light activation is to allow chemical polymerization promoters to react to some extent before being entrapped by the polymeric chains when light activation begins.^10^ In a previous study, delaying the light activation for 2 min compensated for a lower dose of light reaching the cement layer, while no delay was necessary when a high light dose was delivered.^28^ Therefore, the current study approach simplified the comparison of the tested resin cements by using immediate light activation with a standard high-intensity LED curing unit.

In this study, the DC, RP_max_, and gel-time values were obtained without the placement of any indirect restorative material. Consequently, light could directly penetrate into the cement layer. Moreover, using the ATR/FT-NIR technique, the corresponding spectral data could be acquired immediately upon mixing the materials.^21,27^ On the other hand, using this method can make temperature control of the specimen more challenging. It has been reported that the DC of resin cements was significantly affected by the temperature, particularly for resin cements tested in the self-cure activation mode.^18^ Therefore, the use of specimens at a room temperature of 23°C could be a limitation in the current study. Under similar testing conditions, the DC values ranged from 54% to 58.5%, with the exception of MLN, at 76.7%. It has been reported that the DC of dual-cure resin cements is likely material/brand-related, regardless of their category,^10^ in agreement with the results obtained in this study.

The setting process of conventional and self-adhesive resin cements is based on free-radical polymerization. Polymerization activation depends on the photoinitiator (ie, camphorquinone) and the chemical initiator’s molecule (ie, benzoyl peroxide).^10^ Both initiators function with a co-initiator, which is usually a tertiary amine compound.^37^ Acidic monomers present in adhesives or self-adhesive cements may chemically interfere with the amine initiator, triggering incomplete polymerization and decreasing the DC.^17^ Conversely, in the current study, self-adhesive resin cements showed DC values similar to those of conventional resin cements (with the exception of MLN) evaluated without their adhesives. Based on the results obtained in the current study, a different initiation system with a more acid-compatible nature might have been employed in the self-adhesive resin cements tested.

Owing to the limited information available regarding the material formulation, the exact role of each component is unclear. Therefore, aspects of resin cement composition, such as resin matrix, filler content, and initiator system should be carefully considered. In addition, the proportional composition may also differ across the various resin cements, resulting in different polymerization behaviors. For instance, the resin matrix itself contains a mixture of resin monomers with different viscosities and reactivities. When a dual-cure resin cement is light cured, the conversion of the monomers into the polymer begins, and the viscosity of the material increases. The increased viscosity may interfere with the movement of the chemical components responsible for the additional polymerization set.^1^ In addition, the filler content may also restrict the mobility of the monomers, leading to lower DC values.^11,29^ A previous study found that UDMA and bis-EMA present higher DCs than does bis-GMA, but lower DCs than TEG-DMA.^18,31^ The higher flexibility of TEG-DMA increases the mobility during network formation, which increases the DC and cross-link density of the polymer.^31,34^ The higher DC value observed for MLN could be partially explained by the presence of low-viscosity monomers, such as UDMA, bis-EMA and HEMA in its composition.

RP_max_ may provide important information regarding the development of the polymerization process to the final structural network, which consequently affects the mechanical properties of the material.^3^ Cements with relatively low amounts of photosensitizers may polymerize at a slower rate, allowing better arrangement of the polymer matrix and reducing the polymerization stress.^6,14^ Considering the self-adhesive resin cements tested, PSA showed significantly lower RP_max_, with longer gel time and slightly lower shrinkage stress values, suggesting that it is less sensitive to light activation. Similarly, for the conventional resin cements tested, the lower RP_max_ values observed for PNV may have contributed to the lower amount of shrinkage stress. The highest RP_max_ observed for MLN may indicate that it is more sensitive to light activation; therefore, a higher shrinkage stress is expected. Nevertheless, no significant difference in stress values was observed between MLN and RUL.

In this regard, several factors might be involved in stress generation, such as the formulation of the material, reaction kinetics, DC, viscoelastic behavior, and substrate compliance.^10,38^ Considering that the tooth structure has a relatively high compliance, the shrinkage stress test was performed with PMMA rods. Although PMMA rods have been suggested as a better alternative compared to low compliance systems, such as steel or glass rods,^20^ they may yield light attenuation of approximately 30% during polymerization.^14^ In addition, a standard 0.8-mm-thick resin cement was used during the shrinkage stress test, which may not correspond to the results in the clinical situation. Some dual-cure resin cements have been reported to be more dependent on light activation than others.^6^ Thus, light attenuation might have influenced the behavior of resin cements during the stress test.

The stress generated during polymerization may affect the bond strength between the restorative material and the tooth.^36^ However, in this study, the similar shrinkage stress values observed for both categories of resin cements did not reflect the bond strength performance; according to the µTBS results, the use of a dedicated adhesive clearly provided a higher bond strength for all conventional resin cements. Among the cements tested, only MLN is known to lack 10-MDP monomer in its bonding agent. Several studies have reported that 10-MDP may contribute to the formation of a hydrolytically stable bond with calcium via chemical interaction with hydroxyapatite.^22,40^ This could explain the good bonding performance observed for RUL and PNV after both storage periods. As a result, failure between the resin cement and adhesive was mainly observed for those materials. In contrast, for MLN, the initially high bond strength may be attributed to its micromechanical interlocking ability and interaction with superficial dentin, as long resin tags could be observed in the SEM images. Nevertheless, the absence of 10-MDP may have accelerated the degradation process of MLN. Moreover, water storage may reduce the mechanical properties of the polymer matrix.^9^ This may have influenced the increase in the rate of mixed failures found for MLN after 1 year of storage.

In the current study, lower bond strengths were observed for all self-adhesive resin cements tested in comparison with those of the conventional resin cements. The reduced bond strength of self-adhesive resin cements is often associated with their limited potential to demineralize and infiltrate the underlying dentin.^26^ Owing to the higher viscosity of the resin cements compared with that of the bonding agents, monomer diffusion into the dentin tubules may be reduced.^25^ In agreement with these findings, in this study, interfacial images of the self-adhesive resin cements examined showed thinner resin tag formation, particularly for PSA. After short-term storage, the high number of failures at the cement-dentin interface confirmed the initial weakness of interfacial bonding for all self-adhesive resin cements. Nevertheless, stable bonding performance was observed for this cement category after 1 year of storage.

Based on the results obtained in this study, in addition to good bonding performance, the polymerization kinetics and shrinkage stress of the resin cements must be considered for the long-term success of indirect restorations. Despite the limitations of this in vitro study, the results obtained are expected to provide further insights for estimating the clinical performance of resin cements.

## CONCLUSION

Self-adhesive resin cements may not perform as well as the conventional resin cements. Although both categories of cements presented similar polymerization kinetics and shrinkage values, the self-adhesive resin cements showed lower µTBS than those of the conventional resin cements studied. Nevertheless, the storage time only affected the bonding performance of MLN.

## Clinical relevance:

Self-adhesive resin cements may not perform as well as the conventional resin cements. Although both categories of cements presented similar polymerization kinetics and shrinkage values, the self-adhesive resin cements showed lower μTBS than those of the conventional resin cements studied.
